# Appropriate Antibiotic Use and Associated Factors in Vietnamese Outpatients

**DOI:** 10.3390/healthcare9060693

**Published:** 2021-06-08

**Authors:** Lam V. Nguyen, Lien T. T. Pham, Anh L. Bui, Mai T. Vi, Nguyet K. Nguyen, Tam T. Le, Suol T. Pham, Phuong M. Nguyen, Thao H. Nguyen, Katja Taxis, Thang Nguyen, Hung D. Tran

**Affiliations:** 1 Department of Anatomy, Can Tho University of Medicine and Pharmacy, Can Tho City 900000, Vietnam; nvlam@ctump.edu.vn; 2 Department of Medicinal Chemistry, Can Tho University of Medicine and Pharmacy, Can Tho 900000, Vietnam; pttlien@ctump.edu.vn; 3 Department of Pharmacology and Clinical Pharmacy, Can Tho University of Medicine and Pharmacy, Can Tho City 900000, Vietnam; lanabui2510@gmail.com (A.L.B.); maivivi127@gmail.com (M.T.V.); kimnguyetnguyen@gmail.com (N.K.N.); ptsuol@ctump.edu.vn (S.T.P.); nthang@ctump.edu.vn (T.N.); 4 Can Tho University of Medicine and Pharmacy Hospital, Can Tho City 900000, Vietnam; lttam.bv@ctump.edu.vn; 5 Department of Pediatrics, Can Tho University of Medicine and Pharmacy, Can Tho City 900000, Vietnam; nmphuong@ctump.edu.vn; 6 Department of Clinical Pharmacy, University of Medicine and Pharmacy at Ho Chi Minh City, Ho Chi Minh City 700000, Vietnam; huongthao0508@gmail.com; 7 Groningen Research Institute of Pharmacy, University of Groningen, 9713 AV Groningen, The Netherlands; k.taxis@rug.nl; 8 Faculty of Nursing, Can Tho University of Medicine and Pharmacy, Can Tho City 900000, Vietnam

**Keywords:** appropriate antibiotic usage, associated factors, antibiotics, outpatients, Vietnam

## Abstract

*Background*: Inappropriate antibiotic use among outpatients is recognized as the primary driver of antibiotic resistance. A proper understanding of appropriate antibiotic usage and associated factors helps to determine and limit inappropriateness. We aimed to identify the rate of appropriate use of antibiotics and identify factors associated with the inappropriate prescriptions. *Methods*: We conducted a cross-sectional descriptive study in outpatient antibiotic use at a hospital in Can Tho City, Vietnam, from August 1, 2019, to January 31, 2020. Data were extracted from all outpatient prescriptions at the Medical Examination Department and analyzed by SPSS 18 and Chi-squared tests, with 95% confidence intervals. The rationale for antibiotic use was evaluated through antibiotic selection, dose, dosing frequency, dosing time, interactions between antibiotics and other drugs, and general appropriate usage. *Results*: A total of 420 prescriptions were 51.7% for females, 61.7% with health insurance, and 44.0% for patients with one comorbid condition. The general appropriate antibiotic usage rate was 86.7%. Prescriptions showed that 11.0% and 9.5% had a higher dosing frequency and dose than recommended, respectively; 10.2% had an inappropriate dosing time; 3.1% had drug interactions; and only 1.7% had been prescribed inappropriate antibiotics. The risk of inappropriate antibiotic use increased in patients with comorbidities and antibiotic treatment lasting >7 days (*p* < 0.05). *Conclusions*: The study indicated a need for more consideration when prescribing antibiotics to patients with comorbidities or using more than 7 days of treatment.

## 1. Introduction

Ever since antibiotics were introduced into medicine in the 1940s, resistance to them has also been ascertained and acknowledged [1]. The World Health Organization (WHO) recorded that carbapenem antibiotics do not work in more than half of the patients treated for *Klebsiella pneumoniae* infections due to resistance in some countries [2]. The WHO also figured out that 12.11% of *Staphylococcus aureus* observed were methicillin-resistant and *Escherichia coli* resistant to third generation cephalosporins was detected with 36.0% in 2019 [2]. In 2016–2017, a research project in Vietnam on preserving efficacy of last-line antibiotics indicated a very high rate of resistance to most antibiotics, including carbapenems [3]. According to the Centers for Disease Control and Prevention (CDC), widespread inappropriate prescribing of antibiotics to outpatients has been recognized as the primary driver of resistance patterns and is responsible for more than 2,835,000 deaths each year in the United States [1,4,5]. In 2013, Vietnam became the first country in the World Health Organization’s (WHO) Western Pacific Region to approve a national action plan to combat antimicrobial resistance, in which establishing a national surveillance system for antimicrobial resistance is one of the aims [6]. Since 2015, Vietnam’s Ministry of Health has developed and issued the Instructions for Use of Antibiotics providing treatment recommendations for each disease condition and each prophylaxis case, whereby the principles for the appropriate use of antibiotics were specified (Appendix A) [7,8].

In the United States, studies have described the inappropriate selection of antibiotics and the need for rational antibiotic prescribing practices. Among outpatient antibiotic prescriptions filled, 23.2–40% were inappropriate [9,10,11], and they were more prevalent among adults (25.2%) than among children (17.1%) [10]. A recent study in Japan concluded that, of the 125 antimicrobial drugs used for infection treatment, 60 (48.0%) were unnecessary [12]. At a hospital in Vietnam, Vo Thi Thanh Thuy recorded that, for 100% of prescriptions with appropriate antibiotic choices, 98% of cases used the correct dosage of the antibiotic, but for 2%, the prescription was in a lower dose than recommended [13].

Many factors that may be associated with inappropriate antibiotic use by outpatients can be related to characteristics of prescribing physicians, patients, the environment, or the performance of physical examinations [14,15]. In Vietnam, multiple factors are involved in the spread of antimicrobial resistance in general and the inappropriate use of the antimicrobial drug in particular, such as overcrowded, understaffed hospitals with insufficient infection control; antibiotics were often overprescribed or administered with the incorrect dose or duration [16,17].

The complicated situation of antibiotic resistance has, in recent years, raised great concern among local health facilities. Being a hospital with well-trained healthcare staff and associated with teaching/training activities, the Hospital of Can Tho University of Medicine and Pharmacy provides healthcare services for Southwestern Vietnam, with a large number of patients per day and a variety of clinics. The hospital is consistently attentive in the safe, effective, and appropriate use of drugs, especially antibiotics. Several measures to promote the appropriate use of antibiotics have been surveyed and proposed in the hospital, but these focus mainly on inpatient and prophylactic antibiotics. In this study, for a total evaluation of antibiotic use in outpatient treatment, we identified the rate of the appropriate use of antibiotics and assessed a number of factors associated with inappropriate prescription in this setting.

## 2. Materials and Methods

### 2.1. Setting and Study Population

We conducted a cross-sectional descriptive study to analyze outpatient antibiotic prescriptions in clinics of a hospital in Can Tho City, Vietnam. In the region, this general hospital serves a large number of patients from different regions, with a total of 205,848 outpatients and 12,074 inpatients in the year 2019.

We included outpatient prescriptions with antibiotics and excluded prescriptions (1) with no oral antibiotics (e.g., eye drops and topical antibiotics) and (2) lacking information on the indicated drug. Prescriptions repeated at the re-visits of the sampled patients were also excluded.

### 2.2. Data Sources

Data were extracted from all outpatient prescriptions recorded in the hospital’s prescription database for 6 months (from 1 August 2019, to 31 January 2020) at the Medical Examination Department of the Hospital of Can Tho University of Medicine and Pharmacy. Screening of subjects before collection was based on inclusion and exclusion criteria. The eligible prescriptions from a total of 97,126 outpatient prescriptions during the study period were listed in numerical orders; subsequently, the prescriptions numbered at a defined interval were collected according to the technique of systematic random sampling, until 420 prescriptions were reached. Baseline data included demographic characteristics, health insurance, comorbidities, and characteristics of antibiotic use. Prescriptions lacking information on the prescribed drugs were excluded.

### 2.3. Data Analysis

The sample size was estimated by using the formula for a single proportion with an estimated proportion of antibiotic appropriate use which was 0.847, as reported in a prior study [16], an assumed margin of error of 4%, and a confidence level of 95%, resulting in 312 prescriptions. To minimize the error, the sample size was increased by 20%, whereby at least 375 prescriptions were required. We selected a total of 420 outpatient prescriptions as the samples.

Assessment of the appropriateness of the antibiotic use was according to the following documents, in this order of priority: (1) medication instruction sheet, which was revised and approved by the regulatory authority—Drug Administration of Vietnam; (2) Instructions for Use of Antibiotics of Vietnam’s Ministry of Health, issued with Decision No. 708/QD-BYT, dated 2 March 2015; and (3) Vietnamese National Drug Formulary 2018, which specified the use of each drug substance [7,18]. We evaluated the rationale for antibiotic use by using five indicators, namely antibiotic selection, antibiotic dose, dosing frequency, dosing time of antibiotics, interactions between antibiotics and other drugs, and general appropriate usage. Briefly, the antibiotic selection was appropriate when the indicated antibiotic in the prescriptions was consistent with recommendation (1), (2), or (3); as listed [7,18], no antibiotic was absolutely contraindicated in patients with comorbidities, according to recommendations; and prescriptions did not include more than one antibiotic with the same drug substances or from the same drug class. We considered appropriate a 24-h dose, neither lower nor higher than recommended, and a dosing frequency (number of times of antibiotic use per day) not lower or higher than recommended. The appropriate dosing time of antibiotic use was the same as recommended. We used the Drug Interactions Checker tool (www.drugs.com; accessed on 2 March 2020) to look up interactions between antibiotics with other drugs prescribed and considered interactions labeled “Major” by entering the drug list in the prescriptions into the tool and checking for the interactions. A prescription was generally considered appropriate when all five of the indicators listed above were appropriate. This study also assessed the effects of several relevant factors, including age (≤15 years, 16–59 years, and ≥60 years), gender, health insurance, comorbidities, and duration of antibiotic treatment (≤7 days and >7 days) on appropriate antibiotic use.

### 2.4. Statistical Analysis

Collected data were analyzed by using SPSS 18. Descriptive statistics, such as frequencies and percentages, were included in the analysis. The frequencies of categorical variables of two groups were compared by using the Chi-squared test, with 95% confidence intervals and a *p* < 0.05 level of statistical significance.

We modeled a multivariable logistic regression analysis, assuming the inappropriate usage as the outcome variable and age group, gender, health insurance, comorbidities, and duration of antibiotic treatment as explanatory ones. All five explanatory variables were analyzed in both models of univariable and multivariable analysis. A *p* < 0.05 was considered statistically significant.

## 3. Results

### 3.1. Study Population Characteristics

From a total of 97,126 prescriptions, after screening based on inclusion and exclusion criteria, 852 antibiotic prescriptions were eligible for systematic random sampling and 420 prescriptions were included for this study. Of 420 prescriptions, those for female patients were 51.7%. The mean age was 40.6 years (SD: 18.4). Antibiotics prescribed for outpatient treatment accounted for the highest proportion for the age group 16–59 (77.9%) and lowest for the age group ≥ 60 years (20.2%). In total, 61.7% had health insurance. The largest percentage of patients had one comorbid condition (44.0%), followed by those with no comorbidities (41.0%); the lowest percentage had two comorbidities (4.3%). The prescriptions collected from the ear–nose–throat clinic accounted for the highest proportion (35.7%), and those from the oncology clinic for the lowest (0.5%) (Table 1).

### 3.2. Appropriate Usage of Antibiotics

Of 420 prescriptions, the general appropriate rate of antibiotic use accounted for 86.7%. Of these, 98.3% of the prescribed antibiotics were considered appropriate. Higher-than-recommended antibiotic dose and frequency were found in 9.5% and 11.0%, respectively. Dosing time without adherence to medical recommendations accounted for 10.2%. The rate of antibiotic interactions with other drugs was 3.1% (Table 2). Penicillins are the most commonly prescribed classes of antibiotics, accounting for the highest, at 49.2%. The frequency of prescribed antibiotics is illustrated in Appendix B.

### 3.3. Factors Associated with Inappropriate Antibiotic Usage

Results of the univariable analysis reported no association between age, gender, or health insurance and inappropriate antibiotic usage (*p* > 0.05). The risk of inappropriate antibiotic use increased in patients with comorbidities (OR = 2.30; *p* = 0.009) and duration of antibiotic treatment > 7 days (OR = 3.04; *p* = 0.002), with a statistically significant difference.

Results of multivariable logistic regression analysis indicated an association of comorbidities, and duration of antibiotic treatment, with inappropriate antibiotic usage (*p* < 0.05) (Table 3).

## 4. Discussion

### 4.1. Study Population Characteristics

Antibiotic prescribing in the children and elderly age group was lower than reported rates in a study at in public health facilities in Hoa Nhon district, Binh Dinh province with 46.12% of antibiotic prescriptions was performed on age group ≤15 years and 22.15% belonged to age group ≥60 years [16]. In general, the proportion of patients by age group in this study was different from the current Vietnamese population characteristics with 23.214% and 7.554% for population aged <15 and ≥65, respectively [19,20]. This may be due to the vulnerability of individuals of different ages to infectious diseases and different tendency of seeking for medical services among pediatric patients, adults, and elderly patients as well as the availability of other specialized hospitals such as pediatrics and cardiology hospital which help to allocate the patients in the region. A proportion of antibiotic prescriptions covered by health insurance was recorded with 61.7%, which was lower than coverage and use of health insurance in 2019, at 86.4% [21]. In contrast, the percentage in the US, reported in Michael J. Durkin’s study, was 39.8%, considerably lower than our record [22]. The population using health insurance may also be generally related to the proportion of patients in the age groups and the medical conditions. In other words, patients, especially the elderly, usually tend to use health insurance in treating chronic diseases such as cardiovascular diseases while conditions requiring antibiotic therapy were usually short-term, therefore the proportion of elderly patients using health insurance may be low in comparison with some chronic diseases. Patients with at least 2 comorbidities had a low rate in this study, possibly because the elderly patients in our study were quite low as elderly patients are more likely to suffer from underlying diseases. In some countries, the proportion of women receiving antibiotics was 60–66.9% higher than that in our study, possibly due to differences in the need to seek health care [23,24,25]. In contrast, research by Jia Yin et al. showed prescriptions of antibiotics for men (55.1%) to be higher than for women [26].

### 4.2. Appropriate Usage of Antibiotics

Assessing whether or not the use of antibiotics was appropriate can be related to many factors. In this study, the appropriate usage of antibiotics was evaluated according to whether the prescribed antibiotic followed the recommendation indication or not. We recorded 413 (98.3%) of 420 prescriptions involving antibiotic selection in accordance with recommendations, and none containing antibiotics involving absolute contraindication for patients. In addition, no prescription had more than one antibiotic containing the same active ingredient. The selection of appropriate antibiotics in the current study did not differ from that of the previous study in different hospitals, and all were found to be more than 93% appropriate [13,27]. Nevertheless, in a study at Cho Ray Hospital in Ho Chi Minh City, Nguyen Quoc Binh recorded 44/384 (11.5%) outpatient prescriptions using inappropriate antibiotics, a result that was much higher than our result of 1.7% [28]. E. Past noted that antibiotic therapy was deemed inappropriate in 71 (34.1%) inpatient prescriptions at a 1000-bed university teaching hospital in Austria, with an inappropriate selection of antibiotics given as the most common reason (*n* = 45, 63.4%) [29]. These results suggest that physicians at hospitals in Can Tho city more accurately followed the recommendations of the Ministry of Health of Vietnam and the instructions of the drug manufacturer. Alongside the documents, doctors also needed to consider the actual situation at the facility and matters of epidemiology, as well as local antibiotic resistance. However, a limitation of our study is that the aspect of appropriate choice for using or not using an antibiotic was not evaluated, as the clinical features of patients were not recorded in the source database.

The second factor assessed was the dosage of antibiotics for the successful treatment of infections and reduction in resistance rate. We found that 90.5% of prescriptions had an appropriate dosage; 9.5% prescribed a higher-than-recommended dose, and no one had a lower-than-recommended dose. Vo Thi Thanh Thuy recorded 98% of cases using antibiotics with the appropriate dosage, but 2% with doses lower than recommended [13]. In previous research, the reported proportions of appropriate antibiotic dosage were between 66.9% and 81.5% [27,28].

Third, the selection of dosing frequency was also a significant issue. In some cases, a single dose was appropriate, but due to a higher or lower frequency than recommended, resulting in a 24-h dose not being suitable. In our study, the rate of appropriate dosing frequency was quite high (89.0%); 11.0% of cases were higher than recommended. In addition, in 89.8% of prescriptions, we found the timing of antibiotic use to be correct. According to research at the central general hospital, errors in the time for using antibiotics were the most common error, at a rate of 32.6% [28]. The difference between the disease patterns in each hospital, as well as the different sources of reference, could be among the reasons for the discrepancy between these results. Unlike other drugs, whose recommended dosing times are specific times of the day (morning, afternoon, and evening), it is recommended that most antibiotics be taken with meals (before, during, and after meals). As it is important for patients at home to adhere to instructions on when to take the drugs, these instructions should be as detailed as possible. Our results showed that most of the doctors considered it important to advise patients regarding the time to take drugs.

The fifth factor, drug–drug interaction, is an important problem related to simultaneous use of multiple drugs. In our research, of 420 prescriptions examined, the interaction rate between antibiotics and other drugs was low, at 3.1%. However, only major interactions that are highly clinically significant and avoid combinations were recorded in this study. On the other hand, fewer interactions between antibiotics with the other drugs were acknowledged in comparison with those of cardiovascular drugs in general. In a study at Hue University of Medicine and Pharmacy Hospital, the percentage of prescriptions appearing to have clinically significant interactions was higher than our results, with 6.7%. Furthermore, the reported interaction rate was relatively higher in a study at Cho Ray hospital, Ho Chi Minh City, Vietnam, with 18.7% [28]. Due to the increased likelihood of polypharmacy, drug interactions are rather frequent in cases of multi-morbidity [30], while only 4.3% of prescriptions were performed in patients with >2 comorbidity conditions in our population sample, thus explaining the lower rate of drug interactions in our study. Although a number of interactions provided benefits and responses to treatment, most drug interactions led to undesirable effects. It was necessary for physicians to control adverse interactions when prescribing antibiotics to outpatients.

In the viewpoint of the quantity of antibiotic use, Vietnam ranks 11th in antimicrobial consumption per capita, with 32 Defined Daily Doses per 1000 inhabitants per day and is the second low- and lower-middle income country to appear on the list of global antibiotic consumption by country between 2010 and 2015 [31]. Considering the quality perspective of antibiotic use, based on five criteria for evaluating the rationality of antibiotic prescriptions in outpatient treatment, we found 86.7% of prescriptions to be appropriately indicated. This rate was higher than research results reported in public health facilities in Binh Dinh Province (79.8%) and the study at Cho Ray in Ho Chi Minh City (35.4%) [16,28]. In 2016, a cross-sectional study of 19.2 million people aged 0–64 years in the United States recorded that 23.2% of prescriptions were inappropriate [10]. The inappropriate rate at an emergency department in Australia was reported with 32.7% throughout 2016 [32]. In the Netherlands, a recent study on antimicrobial use in the outpatient clinics showed that among 95% of prescriptions for which a guideline was present, the guideline non-adherence rate was 25.6% in term of antimicrobial agent, dose and duration [33]. However, since the criteria for the appropriate use assessment varied among individual studies, only a general comparison can be generated between study data. In brief, the percentage of appropriate antibiotic prescriptions in this study was higher than in previous studies in the country [16,28], and also higher than in some countries around the world [10,11,12,29,32,33]. However, in our sample population, there were a few elderly (20.2%) and children (1.9%) who received the antibiotics; this also means that vulnerability and multi-morbidity are less likely to affect decision-making on antibiotic prescribing of doctors and might be a supporting reason for the lower rate of inappropriate usage. Since warnings about the current situation of antibiotic resistance, physicians have paid great attention to appropriate antibiotic use, making diagnoses to promote effective treatment, limit side effects, save treatment costs, and especially reduce the antibiotic resistance of bacteria. Moreover, studies have been conducted by using different times, locations, disease patterns, and evaluation criteria.

### 4.3. Factors Associated with Inappropriate Antibiotic Usage

Administration of antibiotics to a patient involves many objective factors, ranging from the patient’s characteristics to the disease condition. Normally, physicians choose antibiotics for therapy depending on two factors: the characteristics of the patient and the pathogenic bacteria. The patient’s characteristics should be scrutinized, such as age, drug-allergy history, liver and kidney function, immunodeficiency status, and comorbidities. Regarding pathogenic bacteria, the type of bacteria causing the disease and their sensitivity to antibiotics should also be considered in the treatment of infections. Decision-making in antibiotic prescribing among doctors in Vietnam is influenced by both disease-related characteristics and individual factors, including acceptable minimum treatment coverage [34]. However, due to limited time, as well as inadequate physical conditions at our facility, in this study, we were able to investigate only a few of the important factors related to inappropriate use of antibiotics.

Using univariate analyses and multivariate logistic regression analysis, we found the association between comorbidities, duration of antibiotic treatment, and inappropriate antibiotic usage to be *p* < 0.05.

The rate of using antibiotics unreasonably with patients with comorbidities was 16.9%, 2.30 times higher than in patients without comorbidities (8.1%). This difference was statistically significant, with OR = 2.30 (1.21–4.36) and *p* = 0.009. In a series of nine studies evaluating comorbidities as a prescribing factor, seven found no association, and only two studies demonstrated an association between the presence of comorbid symptoms and the prescription [15]. For patients with comorbid conditions, the administration of antibiotics could greatly affect the degree of disease and the conditions of the body, as well as associated pathological characteristics. For these reasons, it was difficult for doctors to determine a choice of antibiotics, the combination of antibiotics, the route of administration, and the dose and frequency of antibiotics.

Furthermore, we found that, in outpatients using more than 7 days of antibiotic treatment, the rate of using antibiotics inappropriately was 28.6%; this was 3.04 times higher than the group of patients administered antibiotics within 7 days (11.6%). The difference was statistically significant, with OR (95% confidence intervals) = 3.04 (1.45–6.36) and *p* = 0.002. The duration of antibiotic therapy depended on the purpose for use (prophylaxis or empirical therapy and microbiological outcomes) and the patient’s course [6,18]. In outpatients, antibiotic use was mainly empirical, because microbiological results often took a long time. In mild and moderate infections, results were usually reached after 7 to 10 days, but in cases of severe infections, and infections in areas difficult for antibiotics to penetrate (pericardium, meningitis, and bone-joint), the course of treatment was much longer [30]. This could explain our results: in patients with severe diseases whereby long-term antibiotic treatment was required, it was difficult for physicians to avoid inappropriate prescription of antibiotics.

Regarding age and gender, according to the results of univariate analyses and multivariate logistic regression analysis, we had yet to find an association of these two factors with inappropriate use of antibiotics for outpatients. In fact, the prescription of an antibiotic would depend on the characteristics of each age group. According to Rachel McKay et al. (2016), after they retrieved a total of 2848 abstracts and analyzed 97 included in full-text reviews and 28 meeting full inclusion criteria, the result showed that just one of ten studies exploring gender found a statistically significant association between being male and having higher odds of antibiotic prescription [15]. Among nineteen studies exploring age as a factor, thirteen found a statistically significant association between age and higher odds of antibiotic prescription [15]. Regarding the results of the above study, we found that differences between our study and others were due to the different references in each study. Moreover, the disease patterns of each country were not the same, and the evaluations were also not similar.

Regarding health insurance, this was the most important policy in the Vietnamese social security system, ensuring that all citizens had health care, not for profit, and implemented by the Vietnamese government. In order to limit compensations, the Ministry of Health of Vietnam and the hospitals had proposed treatment regimens for each disease, creating a scientific basis for appropriate drug indications. In addition, hospitals had applied information technology to medical examinations and treatment to assist physicians in prescribing. Specifically, on the hospital management software, all drugs were registered with the number of uses per day, route and dose, thereby providing guidance to support physicians in prescribing according to the correct treatment regimen, whether with or without health insurance [35]. Ultimately, that explained our results. We found no association between health insurance and inappropriate use of antibiotics for outpatients according to the results of univariate analyses and multivariable logistic regression analysis.

Overall, comorbidities and antibiotic use longer than 7 days made the risk of inappropriate antibiotic use among outpatients respectively 2.46 and 2.63 times higher.

For the fight against antimicrobial resistance (AMR) global burden, a crucial health approach providing educational programs for all doctors, especially young doctors, was supported in a study in Italy. As suggested by the study, a specific course and training on antibiotics should be included in the core curriculum of the School of Medicine; meanwhile, an antimicrobial-resistance program should be set up in health districts and hospitals; and the institution of a network on AMR, with the AMR sentinel doctors directly involved in monitoring and evaluating trends in AMR in their health districts and hospital should be set up [36]. Further investments in training on AMR issues and the rapid implementation of antimicrobial stewardship (AMS) are also urgently needed in Vietnam’s situation as in other developing countries.

## 5. Conclusions

The rate of appropriate general antibiotic usage was 86.7%. Patients with comorbidities and antibiotic treatment lasting >7 days had inappropriate rates of using antibiotics respectively higher than 2.46 and 2.63 times, as compared to the other group of patients. Therefore, physicians need to consider more carefully when prescribing antibiotics to patients with comorbidities or when administering antibiotics with a treatment longer than seven days. Further research is needed to examine intervention solutions focusing on antibiotic use in outpatients to ensure safe, effective, and economical use of antibiotics, and to reduce antibiotic resistance. Moreover, the initial steps for promoting training on AMR and implementing AMS in Vietnam should be taken into further consideration.

## Figures and Tables

**Table 1:** Study population’s characteristics.

Demographic Characteristics	Frequency	Rate (%)
Gender		
Male	203	48.3
Female	217	51.7
Age group		
Mean age	40.6 ± 18.4	
≤15 years	8	1.9
16–59 years	327	77.9
≥60 years	85	20.2
Health insurance		
Yes	259	61.7
No	161	38.3
Comorbidities		
No	172	41
1 condition	185	44
2 conditions	45	10.7
> 2 conditions	18	4.3
Clinic		
Ear–nose–throat clinic	150	35.7
General internal medicine clinic	65	15.5
Dental clinic	34	8.1
Orthopedic clinic	31	7.4
Anorectal external clinic	28	6.7
Men’s urology clinic	25	6.0
Family medicine clinic	22	5.2
Respiratory clinic	16	3.8
Clinic of liver and infectious diseases	16	3.8
Dermatology clinic	10	2.4
Eye clinic	9	2.1
Obstetrics and gynecology clinic	6	1.4
Pediatric clinic	6	1.4
Oncology clinic	2	0.5

**Table 2:** Appropriate antibiotic usage.

Antibiotic Usage (*n* = 420)	Frequency	Rate (%)
Antibiotic selection		
Appropriate usage	413	98.3
Inappropriate usage	7	1.7
Dose		
As recommended	380	90.5
Higher than recommended	40	9.5
Dosing frequency		
As recommended	374	89.0
More than recommended	46	11.0
Dosing time		
As recommended	377	89.8
Not according to recommendations	43	10.2
Drug interactions		
Yes	13	3.1
No	407	96.9
General appropriate antibiotics use	364	86.7

**Table 3:** Results of univariable and multivariable analyses and logistic regression analysis for factors associated with inappropriate antibiotic usage.

Factors	Inappropriate Antibiotic Usage		Univariable Analysis		Multivariable Analysis	
	*n*	%	OR	*p*	OR	*p*
			(95% Confidence Intervals)		(95% Confidence Intervals)	
**Age**						
≥60 years	16	18.8	1.71	0.095	1.87	0.063
<60 years	40	11.9	(0.91–3.23)		(0.97–3.62)	
Gender						
Male	29	14.3	1.17	0.579	1.22	0.512
Female	27	12.4	(0.67–2.06)		(0.68–2.18)	
Health insurance						
Yes	28	10.8	0.58	0.054	0.64	0.146
No	28	17.4	(0.33–1.01)		(0.35–1.17)	
Comorbidities						
Yes	42	16.9	2.3	0.009	2.46	0.007
No	14	8.1	(1.21–4.36)		(1.28–4.75)	
Duration of use						
>7 days	12	28.6	3.04	0.002	2.63	0.015
≤7 days	44	11.6	(1.45–6.36)		(1.21–5.74)	

## Data Availability

Data sharing not applicable.

## Appendix A

**Table A1:** General principles for appropriate antibiotic use according to Vietnam’s Ministry of Health.

General Principles of Antibiotic Appropriateness Use	Guidance Summary
Use antibiotics for bacterial infections only	Antibiotics are usually effective against bacteria only (except for a few impacts on protozoa, fungi, and viruses). That is the reason why it is important to determine if a bacterial infection exists before using antibiotics. There are two determination methods: subclinical test (bacterial culture test) and clinical diagnosis.
Appropriate antibiotic selection	The selection of antibiotics is subject to the characteristics of the pathogen and patient, positions of bacterial infections, pharmacokinetics, pharmacodynamics, and drug resistance model.
Appropriate dosage, route of administration and duration	Dosage of antibiotics is subject to the patient’s age, patient’s weight, hepatic and renal function, and the intensity of the illness. Dosage recommended in the literature is only an initial suggestion. There is no standard dosage for each case of severe infections. Prescribing inadequate dosage will lead to treatment failure and an increase in rates rate of drug-resistant bacteria. In contrast, the level of drug in the blood must be maintained as per recommendations to avert toxicity if medicine concentration in the blood, highly virulent bacteria, and narrow-spectrum traits (e.g., aminoglycoside and polypeptide) can be monitored.Oral is the route of administration for convenience, safety, and cheap price. Bioavailability of 50% or more is considered as good, 80% or more is considered that absorption by the oral route is similar to that by intravenous route. In the following cases that the oral route cannot be used, a parenteral route should be administered only: when the gastrointestinal tract is affected; when a high blood level of antibiotic is required with difficulties to achieve orally.The duration of antibiotic treatment for mild and moderate infections is usually 7–10 days. Severe infections (such as sepsis) or tissue infections that are difficult for antibiotics to access (such as meningitis, Osteomyelitis, etc.) the course of treatment is usually longer, sometimes up to 4–6 weeks. However, prolonged treatment should not be used to avoid drug resistance, an increase in the rate of undesirable effects and treatment costs.
Appropriate combination therapy	The purpose of antibiotic combination is to expand the spectrum of activity, enhance the effectiveness of treatment and reduce antibiotic resistance. To obtain an appropriate combination of antibiotics, it is important to understand the properties of the antibiotics so that when combined, a synergistic effect is created, avoiding antagonistic and incompatible effects.
Appropriate antibiotic prophylaxis	Antibiotic prophylaxis is the use of antibiotics to prevent infection or reinfection. Only some prophylactic antibiotics are used in the following cases: prophylaxis in surgery and prevention of rheumatic fever.

## Appendix B

**Table A2:** Frequency of prescribed antibiotics.

Antibiotics	Frequency	Proportion % (*n* = 420)
Penicillins	207	49.2
Amoxicillin + Clavulanic acid	206	49
Amoxicillin	1	0.2
Cephalosporins	139	33.1
Cefuroxime	110	26.2
Cefpodoxime	26	6.2
Cefixime	3	0.7
Quinolones	53	12.6
Levofloxacin	22	5.2
Ofloxacin	18	4.2
Ciprofloxacin	13	3.2
Macrolides	18	4.3
Azithromycin	16	3.8
Spiramycin	2	0.5
Tetracyclines	15	3.6
Tetracycline	9	2.2
Doxycycline	6	1.4
